# Early life exposure to structural sexism and late‐life memory trajectories among black and white women and men in the United States

**DOI:** 10.1002/alz.14410

**Published:** 2024-12-18

**Authors:** Justina F. Avila‐Rieger, Paris B. Adkins‐Jackson, Tanisha G. Hill‐Jarrett, Whitney R. Robinson, Katherine M. Keyes, Nicole Schupf, Adam M. Brickman, Richard P. Mayeux, Jennifer J. Manly

**Affiliations:** ^1^ Taub Institute for Research on Alzheimer's Disease and the Aging Brain College of Physicians and Surgeons Columbia University New York New York USA; ^2^ Gertrude H. Sergievsky Center College of Physicians and Surgeons Columbia University New York New York USA; ^3^ Department of Neurology College of Physicians and Surgeons Columbia University New York New York USA; ^4^ Department of Epidemiology Columbia University Mailman School of Public Health New York New York USA; ^5^ Department of Sociomedical Sciences Columbia University Mailman School of Public Health New York New York USA; ^6^ Department of Neurology Memory and Aging Center University of California San Francisco San Francisco California USA; ^7^ Global Brain Health Institute University of California San Francisco San Francisco California USA; ^8^ Trinity College Dublin Dublin Ireland; ^9^ Division of Women's Community and Population Health Department of Obstetrics and Gynecology Duke University School of Medicine Durham North Carolina USA

**Keywords:** intersectionality, memory decline, sex/gender, structural sexism

## Abstract

**INTRODUCTION:**

We investigated whether early life exposure to state‐level structural sexism influenced late‐life memory trajectories among United Staes (U.S.) ‐born women and men and determined whether associations differed between racialized groups.

**METHODS:**

Participants were from the Washington Heights‐Inwood Columbia Aging Project (WHICAP; *N *= 2314) and Health and Retirement Study (HRS; *N *= 18,631). State‐level structural sexism was measured via U.S. census and administrative data and linked to participants in each study by birth year and state.

**RESULTS:**

Exposure to greater structural sexism was associated with lower baseline memory performance among WHICAP women and HRS men and faster memory decline among women in both studies. Women born in the state with the highest structural sexism showed memory decline like that of those who were 9 years older. Structural sexism‐baseline memory associations were stronger among Black women than White women.

**DISCUSSION:**

Early life exposure to structural sexism negatively impacts late‐life memory trajectories among women.

**Highlights:**

- A longitudinal measure captured state‐level structural sexism from 1900 to 1960.
- Exposure to structural sexism was associated with worse late‐life memory outcomes.
- Associations were strongest among women for memory decline.
- The negative impact on memory performance was stronger among Black women.
- Lowering structural sexism may, in turn, reduce memory decline among women.

## 1 BACKGROUND

Women are disproportionately affected by the Alzheimer's disease (AD) epidemic in the United States (U.S.); because women outlive men, they represent nearly two‐thirds of Americans currently living with AD and account for approximately 60% of AD caregivers.^1^ Research aimed at identifying AD risk and protective factors among women has focused mainly on sex‐linked biological mechanisms (i.e., sex‐steroid hormones).^2^, ^3^ However, women in the United States, particularly those aged 65 and older, experience myriad structural barriers throughout their lives. Macro‐level societal inequalities that are rooted in sexism ultimately generate health inequalities^4^ that may negatively impact cognitive aging trajectories and increase the risk for AD. *Structural sexism* refers to the systemic sex/gender discrimination that is fostered within the fundamental economic, social, political, and cultural institutions of our society.^5^ Systemic oppression of women is manifested by economic inequity, underrepresentation of women in government, and discriminatory social policies restricting women's bodily autonomy.^6^ These upstream macro‐level inequalities shape individual health outcomes by creating barriers to health‐enhancing opportunities and resources.^7^, ^8^ Research linking structural sexism to individual health can inform the development of policy and structural interventions to decrease sex/gender inequalities and improve health outcomes among women.

Little is known about the relationship between structural sexism and late‐life cognitive health outcomes. There is, however, a growing body of research exploring the consequences of state‐level structural sexism on other related health outcomes. This work conceptualizes structural sexism as an index of state‐level sex/gender disparities in labor force participation, wages, poverty, and government representation, the presence of reproductive rights (e.g., proximity to abortion providers), and the prominent ideologies influencing gender attitudes (e.g., percentage of a state population composed of religious conservatives). These studies have found that exposure to greater structural sexism is associated with higher mortality rates,^9^ poorer physical functioning and increased risk of chronic health conditions,^5^ unhealthy coping behaviors like disordered eating,^10^ and less accessible and affordable healthcare for women.^6^ To date, this body of work has focused mainly on measuring exposure to structural sexism during adulthood; therefore, there is a need to investigate the health consequences of early life exposure. Early life may be a critical period for structural inequality to have direct or indirect consequences that accumulate over time. Eventually, these exposures produce disparities in chronic physical health conditions that directly influence biomarkers of brain health, the onset of cognitive impairment/decline, and, ultimately, dementia.^11^

For women racialized as Black, the consequences of structural sexism are intertwined with structural racism. An intersectionality perspective posits that systems of oppression (e.g., sexism, racism) overlap and interact with each other to shape social inequality and individual health outcomes depending on one's social identity or position (e.g., sex/gender, race).^12^ Thus, the effects of structural sexism and racism are multiplicative, that is, they vary as a function of each other, such that the negative impact of one can be amplified by the other. Indeed, two recent studies found that exposure to state‐level structural sexism disproportionately impacted health outcomes among Black and Latinx women compared with White women.^6^, ^13^ There is evidence of heterogeneity in late‐life cognitive health outcomes by racialized group, with Black women showing faster rates of cognitive decline^14^ and higher rates of incident dementia^15^ compared to their non‐Latinx White counterparts. Identification of potential upstream drivers of this heterogeneity is essential for the development of structural interventions to improve late‐life cognitive health among women.

The purpose of this study was to examine the association between exposure to state‐level structural sexism and memory trajectories among U.S.‐born women and men and determine whether associations differ between racialized groups. We hypothesized that early life exposure to higher state‐level structural sexism would be associated with lower memory performance and a more rapid rate of memory decline among women, with no association present among men. We also expected the negative impact of structural sexism to be stronger among Black women compared with White women.

## 2 METHODS

### 2.1 Study design, participants, and setting

Analyses examined individual participant data from two longitudinal cohort studies: the Washington Heights‐Inwood Columbia Aging Project (WHICAP)^16^ and the Health and Retirement Study (HRS).^17^ WHICAP is an ongoing study of community‐dwelling Medicare‐eligible people 65 years and older residing in northern Manhattan, New York. HRS is a nationally representative longitudinal study of Americans 50 years and older (details for each study are in the Supplemental Methods).

We included participants who reported their racialized group as either non‐Latinx Black or non‐Latinx White, were U.S.‐born, and dementia‐free at their baseline cognitive assessment. WHICAP participants were born between 1902 and 1954. To create similarity between the two study samples, we excluded HRS participants who were born before 1900 or after 1960 and those who were lost to attrition before age 65. Since HRS waves before 1996 did not use the same memory tests as subsequent waves, we only used visits between 1996 and 2020. We also excluded participants who dropped out or died before the 1996 wave.

### 2.2 Measurements

#### 2.2.1 Structural sexism measure

Building on previous work,^5^ we compiled state‐level indicators representing sex/gender disparities in access to resources and social mobility (i.e., economic, political, cultural, and reproductive health disparities) from the years 1900 to 1960. State‐level data were extracted from publicly available data sources (Table S1). Specific indicators included men‐women ratios for labor force participation, median weekly earnings of full‐time wage and salary workers, percent above the poverty threshold, and state legislature seats, as well as the population composed of religious conservatives and the maternal mortality ratio (maternal deaths per live births). Detailed information on indicator selection and data acquisition is provided in the Supplemental Methods.

#### 2.2.2 Moderators

Participants were given a brief list of racialized groups to identify with based on the 1990 U.S. Census guidelines which included White, Black, Asian, American Indian, Pacific Islander, or other. Participants were asked whether they identified as Hispanic or Latino, and male or female. We will use the term “sex/gender” because it is unknown whether participants actually reported their sex or their gender.

**RESEARCH IN CONTEXT**

1. **Systematic review**: PubMed and Google Scholar databases were searched to identify scientific articles on structural sexism in relation to health outcomes, including cognitive health (i.e., cognitive function, cognitive decline, and dementia).
2. **Interpretation**: Early life exposure to state‐level structural sexism was associated with worse late‐life cognitive health outcomes. These associations were strongest among women for memory decline. Women born in the state with the highest structural sexism showed memory decline similar to those who were 9 years older in age.
3. **Future directions**: To the best of our knowledge, this study is the first to investigate associations between structural sexism and late‐life cognitive health outcomes. Additional studies are necessary to understand the impact of lifecourse exposure to structural sexism on late‐life cognitive decline. Future studies should also examine interactions between structural sexism and structural racism to determine the differential impact of these structural determinants on cognitive health among women racialized as Black.

#### 2.2.3 Covariates

Participant‐level covariates included age at first cognitive assessment and time to death. We did not adjust for individual‐level socioeconomic factors (i.e., educational attainment, occupational attainment, income) because these factors are downstream consequences of structural sexism and not confounders.

We included four time‐varying state‐level covariates based on state of birth: inflation‐adjusted median income (relative to 1960), unemployment rate (capturing economic opportunity), Gini coefficient (measuring income inequality), and proportion of White state residents.

#### 2.2.4 Memory performance

Memory was assessed via immediate and delayed word list recall measures in both studies. WHICAP, assessed memory performance with the immediate, delayed, and recognition trials from the Selective Reminding Test (SRT).^18^ Each variable was converted to z‐scores using means and standard deviations from the entire WHICAP sample at their baseline assessment. Composite scores were then computed by averaging the z‐scores at each study visit. In HRS, memory was assessed using the immediate and delayed recall trials of the CERAD^19^ 10‐item word list memory test. We used the cognitive scores provided by HRS RAND, which included imputed cognitive scores for those who participated in a given wave but did not have cognitive data for that wave. Composite scores were computed as they were in the WHICAP sample. Compared with the CERAD, the SRT has a longer word list, more learning trials, and a recognition trial. These differences prevent statistical comparison of composite scores and model parameters across studies.[Fig alz14410-fig-0001]

### 2.3 Statistical analysis

The construction of the structural sexism measure involved several steps. First, confirmatory factor analysis (CFA) was used to estimate structural sexism as a latent factor for each decennial year from 1900 to 1960. Model fit was assessed via the root mean square error of approximation (RMSEA < 0.08), standardized root mean square residual (SRMR < 0.08), Tucker–Lewis index (TLI > 0.90), and comparative fit index (CFI ≥ 0.95).^20^ Next, approximate measurement invariance across time was determined using cross‐classified factor analysis.^21^ Finally, factor scores were generated for each decennial and intercensal year between 1900 and 1960 using a two‐level time series analysis with a first‐order autoregressive CFA via Mplus.^22^ Factor scores were linked to individual HRS and WHICAP participants by year and state of birth.

Memory trajectories (level of memory performance and rate of decline over 2–14 follow‐up assessments) were characterized by estimating separate known‐class mixture models for each study, with sex/gender groups as the known‐class grouping variable. This known grouping variable is incorporated into these models as a moderator variable, allowing model parameters to vary as a function of membership in the identified groups.^23^ Time was parameterized as years from participants’ first cognitive assessment, divided by 10i. Model intercepts indicate baseline memory performance, and slopes indicate change in memory scores per decade. Joint modeling, which combines a latent growth model with a survival model, was used to account for the influence of differential attrition due to death on cognitive trajectories. A retest spline was also included to account for practice effects. Initial models examined associations between structural sexism and memory trajectories across women and men while adjusting for baseline age, race, and state‐level covariates on baseline memory and rate of memory decline (Model 1). Next, interaction terms were included to examine associations by racialized group (Model 2). For each model, robust standard errors were clustered by state of birth. We tested sex/gender differences in associations between structural sexism and memory growth factors using the “Model Constraint” option in Mplus version 8.6.^22^ Both *p*‐values and confidence intervals were used to determine statistical significance.

## 3 RESULTS

### 3.1 Confirmatory factor analysis

**FIGURE 1 alz14410-fig-0001:**
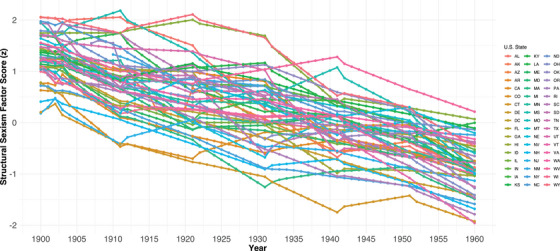
Structural sexism factor scores by state and year from 1900 to 1960. Factor scores were generated for each decennial and intercensal year between 1900 and 1960 using a two‐level time series analysis with a first‐order autoregressive confirmatory factor analysis.

Model fit indices for each decennial year are presented in Table S2. Decennial years 1900, 1910, 1940, and 1960 consistently achieved good model fit across parameters, while 1920, 1930, and 1950 met parameters for TLI and SRMR only. Standardized factor loadings were above 0.30 for all indicators across decennial years. The highest loading indicator was Poverty for 1900, 1920, 1940, and 1960, and Religious Conservatives was the highest for 1910, 1930, and 1950. The lowest loading indicator was Earnings in 1900, Legislature Seats in 1910, 1920, 1930, and 1960, and Labor Force Participation in 1940 and 1950. Approximate measurement invariance analyses met criteria for metric invariance (all factor load variances < 0.01) and partial scalar invariance (all item intercepts, except for labor force participation, were < 0.04). Figure 1 shows generated factor scores by State for every year between 1900 and 1960. Levels of structural sexism vary across states within each year, and there is also a general decline in structural sexism over time. The distribution of scores has a mean of 0 and a standard deviation (SD) of 1. Not all states and years between 1900 and 1960 are represented in WHICAP and HRS. While the distribution of factor scores varied across studies (WHICAP: M = ‐0.120, SD = 0.74; HRS: M = 0.019, SD = 0.62), rather than center at study‐specific values, we keep scores centered at their original distribution (M = 0, SD = 1) in analytic models to facilitate interpretation of findings. Within both studies, the state with the highest structural sexism score was Mississippi in 1910, and the lowest was Connecticut in 1940.

### 3.2 Participant characteristics

Derived samples for each study are presented in Figure S1. Participant characteristics are presented in Table 1 by study, sex/gender, and racialized group. Approximately 77% of WHICAP participants and 89% of HRS participants completed two or more cognitive assessments. The average number of years in each study was 4.7 (SD = 4.5) for WHICAP and 8.5 (SD = 6.4) for HRS.

**TABLE 1:** Baseline characteristics of each study sample.

*WHICAP sample*	Total	White men	White women	Black men	Black women
Characteristic	(*N* = 2314)	(*N* = 336)	(*N* = 462)	(*N* = 451)	(*N* = 1065)
Age, mean (SD)	75.1 (6.3)	74.1 (5.6)	74.4 (5.9)	74.3 (6.2)	75.9 (6.5)
Birth year, mean (SD)	1928 (11)	1931 (10)	1929 (11)	1929 (12)	1927 (11)
Birth year, range	1902–1954	1904–1954	1904–1950	1903–1954	1902–1954
Study years, mean (SD)	4.8 (4.5)	4.5 (4.3)	5.1 (4.6)	4.2 (4.3)	4.9 (4.3)
Years of education, mean (SD)	12.9 (3.8)	15.1 (3.7)	14.6 (3.1)	11.7 (3.7)	11.9 (3.5)
Birth region					
Northeast	1218 (53%)	262 (78%)	368 (80%)	188 (42%)	400 (38%)
Midwest	139 (6%)	42 (12%)	48 (10%)	16 (4%)	33 (3%)
South	933 (40%)	25 (7%)	37 (8%)	246 (55%)	625 (59%)
West	24 (1%)	7 (3%)	9 (2%)	1 (1%)	7 (1%)
Structural sexism, mean (SD)	−0.120 (0.7)	−0.451 (0.5)	−0.414 (0.5)	−0.073 (0.8)	.095 (0.8)

*HRS sample*	Total	White men	White women	Black men	Black women
Characteristic	(*N* = 18631)	(*N* = 6845)	(*N* = 8813)	(*N* = 1150)	(*N* = 1823)
Age, mean (SD)	69.9 (6.7)	69.5 (6.0)	69.9 (6.5)	68.3 (5.2)	68.0 (5.3)
Birth year, mean (SD)	1934 (12)	1934 (11)	1933 (12)	1938 (11)	1938 (11)
Birth year, range	1900–1956	1900–1956	1900–1956	1900–1956	1903–1956
Study years, mean (SD)	8.3 (6.4)	8.3 (6.4)	8.9 (6.4)	6.8 (5.9)	7.7 (6.2)
Years of education, mean (SD)	12.6 (2.9)	13.1 (2.8)	12.7 (2.4)	11.5 (3.4)	12.0 (2.9)
Birth region					
Northeast	4177 (22%)	1751 (26%)	2151 (24%)	111 (10%)	164 (9%)
Midwest	6056 (33%)	2579 (38%)	3217 (37%)	89 (8%)	171 (9%)
South	6894 (37%)	1873 (27%)	2646 (30%)	923 (80%)	1452 (80%)
West	1504 (8%)	642 (9%)	799 (9%)	27 (2%)	36 (2%)
Structural sexism, mean (SD)	0.019 (0.6)	−0.005 (0.6)	0.027 (0.6)	0‐0.003 (0.6)	−0.039 (0.6)

*Note*: WHICAP is the “Washington Heights‐Inwood Columbia Aging Project,” and HRS is the “Health and Retirement Study”.

Abbreviation: SD, standard deviation.

Black women in WHICAP were older at baseline compared with Black men and White women and men. In HRS, Black women and men were younger than White participants at their first study visit. In both studies, more than half of the Black participants were born in a southern state. Average structural sexism scores at birth were similar among White women and men in WHICAP and lower than scores for Black women and men. Black women in WHICAP were exposed to higher levels of structural sexism compared with Black men. White women in HRS were exposed to the highest levels of structural sexism at birth, followed by White and Black men, who had higher scores compared with Black women.

### 3.3 Associations between structural sexism and memory trajectories

Estimates from Model 1 (Table 2) show that early life exposure to higher levels of structural sexism was associated with lower baseline memory performance among WHICAP women and more rapid memory decline among women in both studies. To put these results into perspective, we compared associations between structural sexism and memory decline to associations between age and memory decline. The difference in the rate of memory decline between being born in the state with the highest structural sexism (Mississippi in 1910) versus the state with the lowest structural sexism (Connecticut in 1940) was equivalent to 9.1 (WHICAP) to 9.6 (HRS) years. In other words, women born in the highest structural sexism state had memory decline similar to those who were about 9 years older in age (see Table S3 for age comparisons).

**TABLE 2:** Associations of structural sexism with baseline memory performance and memory decline across sex/gender and study.

	WHICAP		HRS	
	Baseline memory	Rate of memory decline	Baseline memory	Rate of memory decline
Parameter	β (95% CI)	β (95% CI)	β (95% CI)	β (95% CI)
**Women**				
*Intercept*	*.193 (0.11, 0.27)*	*−0.877 (−0.95, −0.21)*	*.209 (0.15, 0.27)*	*−0.654 (−0.68, −0.63)*
Structural sexism	−0.113 (−0.21, −0.02)	−0.191 (−0.28, −0.10)	−0.020 (−0.07, 0.03)	−0.085 (−0.13, −0.04)
**Men**				
*Intercept*	*−0.033 (−0.15, 0.09)*	*−0.919 (−1.02, −0.82)*	*−0.114 (−0.19, −0.04)*	*−0.579 (−0.61, −0.55)*
Structural sexism	−0.051 (−0.19, 0.09)	−0.109 (−0.30, 0.08)	−0.058 (−0.12, 0.01)	−0.032 (−0.07, 0.01)
**Sex/gender difference**				
Structural sexism	−0.062 (−0.25, −0.13)	−0.082 (−0.28, 0.12)	.038 (−0.02, 0.09)	−0.053 (−0.10, −0.01)

*Note*: Intercept is the average baseline memory performance and rate of memory decline for individuals with reference values for all covariates. Rate of memory decline represents change in memory scores per decade. Beta coefficients for structural sexism represent the change in baseline memory and memory decline per 1‐unit increase in the structural sexism measure. WHICAP is the “Washington Heights‐Inwood Columbia Aging Project,” and HRS is the “Health and Retirement Study.”

Abbreviation: CI, confidence interval.

Negative associations between structural sexism and memory trajectories were also present among men in both studies (Table 2); however, these estimates were not significantly different from zero (*p* > 0.05). In both studies, the magnitude of association between structural sexism and baseline memory performance was similar among women and men (sex/gender difference in parameter estimates: HRS B = 0.038, 95% CI −0.02, 0.09, *p* = 0.18; WHICAP B = −0.062, 95% CI −0.25, 0.13, *p* = 0.86). In HRS, the association between structural sexism and memory decline was stronger among women than men (B = −0.053, 95% CI −0.10, −0.01, *p* = 0.003). While a similar pattern was present in WHICAP, estimates for men and women were not reliably different from each other (B = −0.082, 95% CI −0.28, 0.12, *p* = 0.64). It should be noted that the confidence intervals surrounding estimates for WHICAP men are large, suggesting a lack of precision.

**FIGURE 2:**
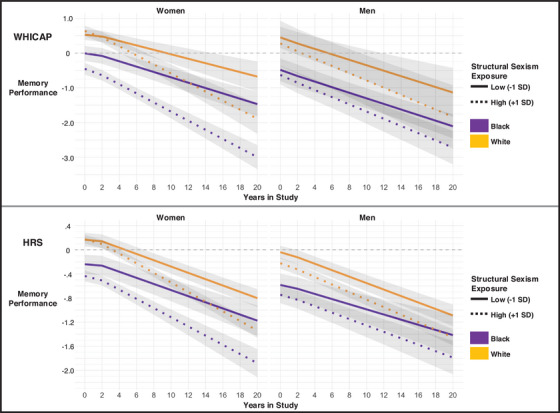
Predicted memory trajectories at low and high levels of structural sexism across racialized groups, sex/gender, and study. Figure 2 plots predictions from Model 2 for individuals at ± 1 SD from the overall mean of structural sexism in the United States between 1900 and 1960. The x‐axis represents the years since the participants’ baseline visit, and the y‐axis represents the corresponding predicted memory score. The figure shows that, in both studies, associations between structural sexism and baseline memory performance were stronger among Black women compared with White women. Women in both studies also showed stronger structural sexism‐memory decline associations compared with men.

Model 2 included race by structural sexism interactions on baseline memory performance and memory decline (Table 3; Figure 2). In both WHICAP and HRS, associations between structural sexism and baseline memory performance were stronger among women racialized as Black than women racialized as White. Black women exposed to higher levels of structural sexism demonstrated lower baseline memory performance compared with their counterparts exposed to lower levels of structural sexism (Model 2 simple slopes: HRS b = −0.065, 95% CI −0.12, −0.01; WHICAP b = −0.144, 95% CI −0.24, −0.05). Among White women, baseline memory performance was similar across levels of structural sexism (HRS b = 0.003, 95% CI −0.05, 0.05; WHICAP b = 0.036, 95% CI −0.12, 0.19). Exposure to higher levels of structural sexism was reliably associated with faster declines in memory performance among Black (HRS b = −0.070, 95% CI −0.13, −0.01; WHICAP b = −0.189, 95% CI −0.32, −0.06) and White women (HRS b = −0.091, 95% CI −0.13, −0.05; WHICAP b = −0.228, 95% CI −0.43, −0.03). These associations did not differ across Black and White women in either study (Table 3).

**TABLE 3:** Interaction effects of structural sexism and race on baseline memory performance and memory decline across sex/gender and study.

	WHICAP		HRS	
	Baseline memory	Rate of memory decline	Baseline memory	Rate of memory decline
Parameter	β (95% CI)	β (95% CI)	β (95% CI)	β (95% CI)
**Women**				
Race	−0.812 (−0.95, −0.67)	−0.069 (−0.28, 0.14)	−0.509 (−0.56, −0.46)	.026 (−0.02, 0.07)
Structural sexism	.036 (−0.12, 0.19)	−0.228 (−0.43, −0.03)	.003 (−0.05, 0.05)	−0.091 (−0.58, 0.36)
Race x structural sexism	‒0.180 (−0.33, −0.03)	.039 (−0.22, 0.30)	‒0.069 (−0.12, −0.02)	.021 (−0.05, 0.09)
**Men**				
Race	−0.920 (−1.3, −0.54)	−0.004 (−0.44, 0.43)	−0.533 (−0.55, −0.48)	.101 (0.04, 0.16)
Structural sexism	−0.058 (−0.34, 0.22)	−0.110 (−0.58, 0.36)	−0.061 (−0.10, −0.01)	−0.035 (−0.08, 0.01)
Race x Structural sexism	.009 (−0.26, 0.28)	.012 (−0.44, 0.46)	.007 (−0.07, 0.09)	−0.009 (−0.09, 0.07)

*Note*: Rate of memory decline represents change in memory scores per decade. WHICAP is the “Washington Heights‐Inwood Columbia Aging Project,” and HRS is the “Health and Retirement Study.”

Abbreviation: CI, confidence interval.

Reliable interactions were not present among men (Table 3; Figure 2). Within HRS, simple slopes for baseline memory performance among White men revealed point estimates like that of Black women (b = ‐0.061, 95% CI ‐0.10, ‐0.01); however, this association for Black men was not significantly different from zero (b = ‐0.054, 95% CI ‐0.16, 0.05). In WHICAP, both Black and White men demonstrated negative associations with baseline memory performance that were weaker than the association for Black women and not significantly different from zero (White men b = ‐0.058, 95% CI ‐0.34, 0.22; Black men b = ‐0.049, 95% CI ‐0.19, 0.09). Negative associations between structural sexism and memory decline were present among Black and White men in both studies (HRS White men b = ‐0.035, 95% CI ‐0.08, 0.01; HRS Black men b = ‐0.044, 95% CI ‐0.12, 0.03; WHICAP White men b = ‐0.110, 95% CI ‐0.58, 0.36; WHICAP Black men b = ‐0.098, 95% CI ‐0.32, 0.13). Estimates for these associations were not significantly different from zero and were weaker than those for women. The wide confidence intervals for White men in WHICAP are likely due to both a smaller sample size and limited variation in birth state (approximately 70% were born in New York).

## 4 DISCUSSION

In both a racially diverse community‐based study and a large nationally representative study, we observed that early life exposure to structural sexism negatively impacts late‐life memory trajectories. For women, greater exposure to structural sexism was associated with faster rates of memory decline. The difference in the rate of memory decline between being born in the state with the highest structural sexism versus the state with the lowest structural sexism was equivalent to 9.1 to 9.6 years of cognitive aging. These findings are consistent with previous studies showing that unequal access to sociopolitical and economic resources has a detrimental impact on women's health outcomes.^5^, ^6^, ^9^, ^10^ This work adds to the literature by showing that these macro‐level structural inequalities also influence late‐life cognitive health outcomes.

Taking a lifecourse perspective, exposure to high levels of structural sexism in early life may have direct biological consequences that increase a woman's risk for cognitive decline later in life.^24^ This risk may remain despite exposure to lower levels of structural sexism throughout the rest of the lifecourse. It is also possible that the downstream consequences of structural sexism trigger a trajectory of social exposures (e.g., educational and occupational opportunities, income, etc.,) that alter risk for cognitive decline at later life stages.^11^ Future studies should test these specific pathways to identify the distinct contributions of policy exposures across the life course.

Structural sexism also had cognitive health consequences for men in both studies. While estimates for men were not significantly different from zero, associations between structural sexism and baseline memory performance were similar among men and women. These findings suggest a potential pattern of universal harm associated with exposure to structural sexism.^5^ Cross‐national studies have demonstrated that gender equity is associated with greater economic growth, poverty reduction, and health improvements at the population level.^25^ More research is needed to understand the pathways linking state‐level structural sexism to deleterious cognitive health outcomes among men in the United States. Estimates for men in this study should be interpreted with caution as confidence intervals were wide, suggesting imprecision.

Results from the current study provide further support that structural sexism is uniquely experienced by women racialized as Black. In both studies, women racialized as Black demonstrated reliably stronger associations between structural sexism and baseline memory performance compared to women racialized as White. It is likely that, for women racialized as Black, the simultaneous and intersectional impact of sexism and racism creates a unique form of oppression that has greater salience for cognitive health than sexism or racism alone.^26^ This intersectional oppression also produces greater depression,^27^ more adverse birth outcomes,^28^ increased social isolation,^29^ and greater heart disease risk factors,^30^ as well as higher subjective cognitive complaints.^31^ Future research would benefit from explicating these pathways further.

The current measure of structural sexism does not capture the unique and intersectional structural barriers experienced by women racialized as Black. Black women were denied access to the voting booth until the 1965 Voting Rights Act and have experienced three times the maternal mortality rate of women racialized as White since 1950.^32^ Women racialized as Black also experience exacerbated gender pay gaps where they make 67 cents for every dollar earned by men racialized as White, which is 17 cents less than women racialized White.^33^ It is possible that the current study underestimates the impact of structural sexism among Black women. A tailored measure of their intersectional experience is vital.

While this study focused on structural sexism in state‐level environments, policies and practices at county and local levels may also exert powerful influences that shape health.^34^ Examining structural sexism in these lower geographic areas between 1900 and 1960 was difficult due to changing geographic boundaries over time and a lack of available data. Another limitation was inconsistent model fit across decennial years, which may have introduced measurement issues. These issues were likely due to sample size restrictions due to the geographic boundaries of the United States.

Including the nationally representative HRS sample strengthened the current study by enabling the examination of structural sexism exposure across a more comprehensive representation of birth states and birth years than the WHICAP sample. The distribution of HRS participants within U.S. birth regions is largely consistent with population estimates from 1900 to 1950 (not 1960) across racialized groups.^35^ Nonetheless, since states are not equally represented across participant birth years, the national generalizability of our findings may be limited across time.

Women in the United States aged 65 and older have experienced structural barriers that prevented them from accessing opportunities and resources necessary for cognitive health. This study provides some of the first insights into the relationship between structural sexism and late‐life cognitive aging outcomes. Among U.S.‐born women racialized as Black and White, early life exposure to structural sexism negatively impacts late‐life memory trajectories. Structural sexism is an actionable risk factor for cognitive decline that can be modified through policy changes. A deeper understanding of the pathways linking structural sexism to cognitive decline and AD risk can inform the development of future policy interventions to improve late‐life cognitive health among minoritized groups like women and people racialized as Black, who have a greater AD burden.

## CONFLICT OF INTEREST STATEMENT

None. Author disclosures are available in the supporting information.

## CONSENT STATEMENT

All participants in both WHICAP and HRS provided written informed consent.

## Supporting information



Supporting Information

Supporting Information

Supporting Information

Supporting Information

Supporting Information

Supporting Information
