# Supplementary material for: Early life exposure to structural sexism and late‐life memory trajectories among black and white women and men in the United States
Source: Alzheimers Dement. 2024 Dec 18;21(2):e14410. doi: 10.1002/alz.14410 (PMC11848392; doi:10.1002/alz.14410)
Supplement: Supplementary file 1 — Supporting Information [file ALZ-21-e14410-s004.pdf]

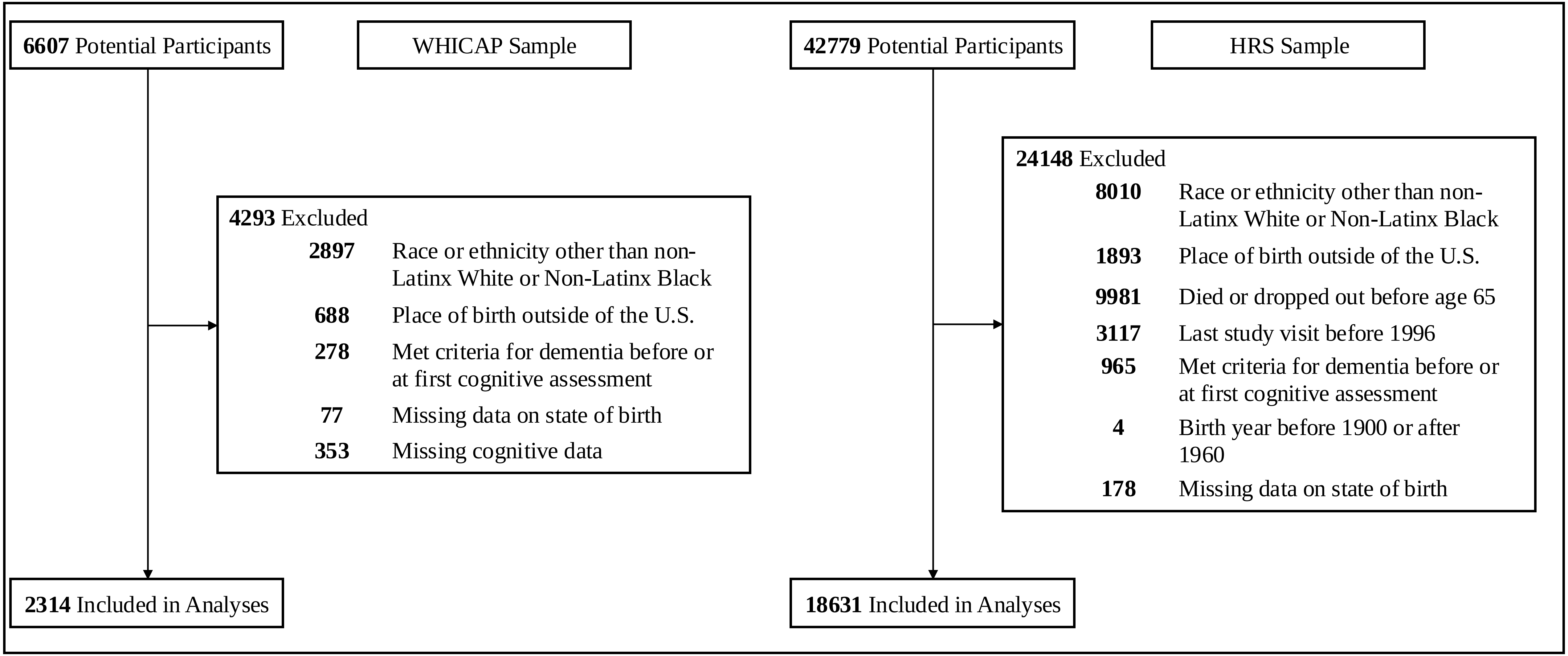

Supplemental Figure 1. Schematic representation of derived samples for the WHICAP and HRS studies.
