# Supplementary material for: Early life exposure to structural sexism and late‐life memory trajectories among black and white women and men in the United States
Source: Alzheimers Dement. 2024 Dec 18;21(2):e14410. doi: 10.1002/alz.14410 (PMC11848392; doi:10.1002/alz.14410)
Supplement: Supplementary file 2 — Supporting Information [file ALZ-21-e14410-s001.pdf]

**Supplemental Table 1.** Measures of state-level structural sexism		
Dimension	Measure	Data Source
Economic	Ratio of men's to women's labor force participation rates (age 16+)	IPUMS USA [1] (author calculation)
	Ratio of men's to women's median weekly earnings (full-time wage and salary workers)	IPUMS USA [1] (author calculation)
	Ratio of men's to women's poverty rate (percent *above* the federal poverty line)	IPUMS USA [1] (author calculation)
Political	Ratio of men's to women's state legislature seats	Women State and Territorial Legislators, 1895-1995 [2]<br>Institute for Public Policy and Social Research (IPPSR) [3]
Cultural	Percent of state population composed of religious conservatives (Evangelical Protestant or LDS)	Association of Religion Data Archives [4]
Reproductive	Maternal Mortality Ratio (maternal deaths per live births)	U.S. Vital Statistics System [5]

References

[1] Ruggles S, Flood S, Sobek M, Backman D, Chen A, Cooper G, et al. IPUMS USA: Version 14.0 2023. https://doi.org/10.18128/D010.V14.0.

[2] Cox EM. Women State and Territorial Legislators, 1895-1995. Jefferson, North Carolina: McFarland & Company Inc; 1996.

[3] Grossman M, Jordan MP, McCrain J. The Correlates of State Policy and the Structure of State Panel Data. State Polit Policy Q 2021:1–21. https://doi.org/10.1017/spq.2021.17.

[4] Gurrentz B, The Association of Religion Dara Archives. History of American Religion, 1770-1930 2020. https://doi.org/10.17605/OSF.IO/4R96U.

[5] Statistics NRC (US) C on N. The U.S. Vital Statistics System: A National Perspective. Vital Stat. Summ. Workshop, National Academies Press (US); 2009.
