# Supplementary material for: Early life exposure to structural sexism and late‐life memory trajectories among black and white women and men in the United States
Source: Alzheimers Dement. 2024 Dec 18;21(2):e14410. doi: 10.1002/alz.14410 (PMC11848392; doi:10.1002/alz.14410)
Supplement: Supplementary file 3 — Supporting Information [file ALZ-21-e14410-s002.pdf]

Supplemental Table 2. Results from confirmatory factor analyses

	Decennial Year						
**Factor Loadings**	**1900**	**1910**	**1920**	**1930**	**1940**	**1950**	**1960**
Indicator							
Religious Conservatives	0.666	0.761	0.675	0.792	0.629	0.736	0.734
Maternal Mortality	0.645	0.583	0.544	0.711	0.761	0.715	0.757
Legislature Seats	0.628	0.446	0.408	0.415	0.465	0.436	0.426
Poverty Rate	0.805	0.615	0.704	0.561	0.691	0.682	0.947
Labor Force Participation	0.742	0.667	0.447	0.67	0.404	0.423	0.48
Earnings	0.624	0.508	0.632	0.605	0.458	0.579	0.454
**Model Fit Statistics**							
N	45	46	48	48	48	48	50
Chi-Square (DF = 9)	9.88	9.88	18.56	17.88	8.59	12.75	7.95
RMSEA	.047	.045	.150	.140	0	.092	0
CFI	0.99	0.985	0.931	0.924	1	0.939	1
TLI	0.983	0.974	0.918	0.917	1	0.919	1
SRMR	0.049	0.112	0.075	0.072	0.058	0.069	0.054

Abbreviations: N, number of states in analyses; RMSEA, root mean square error of approximation; CFI, comparative fit index, TLI, Tucker–Lewis index; SRMR, standardized root mean square residual.
