# Supplementary material for: Early life exposure to structural sexism and late‐life memory trajectories among black and white women and men in the United States
Source: Alzheimers Dement. 2024 Dec 18;21(2):e14410. doi: 10.1002/alz.14410 (PMC11848392; doi:10.1002/alz.14410)
Supplement: Supplementary file 4 — Supporting Information [file ALZ-21-e14410-s006.pdf]

Supplemental Table 3. Results from Model 1 in terms of years of aging

	WHICAP		HRS	
	Baseline Memory	Rate of Memory Decline	Baseline Memory	Rate of Memory Decline
**Women**				
Age, β (95% CI)	-.062 (-.071, -.053)	-.081 (-.097, -.065)	-.051 (-.054, -.049)	-.034 (-.036, -.031)
Difference between highest vs lowest structural sexism state	-.506	-.741	-.076	-.325
Equivalent years of aging (95% CI)	8.2 (1.1, 15.2)	9.1 (4.9, 13.6)	1.5 (-1.6, 4.6)	9.6 (4.9, 13.4)
**Men**				
Age, β (95% CI)	-.058 (-.068, -.048)	-.076 (-.093, -.059)	-.049 (-.052, -.045)	-.025 (-.029, -.022)
Difference between highest vs lowest structural sexism state	-.269	-.450	-.222	-.122
Equivalent years of aging (95% CI)	4.6 (-4.7, 13.9)	5.9 (-3.9, 15.8)	4.5 (0.9, 8.1)	4.9 (-0.3, 10.1)

Note: To estimate the impact of structural sexism in terms of years of aging, associations between structural sexism and memory trajectories were compared with associations between age and memory trajectories. The beta coefficient for age was obtained from separate multiple-group joint models that only included associations of baseline age with baseline memory performance and rate of memory decline. The difference between the highest versus lowest structural sexism state was calculated from Model 1 and represents the difference in predicted baseline memory performance or predicted memory decline between the state with the highest versus lowest structural sexism score. Equivalent years of aging was calculated by dividing the difference between the highest versus lowest structural sexism state by the beta coefficient for age. WHICAP is the “Washington Heights-Inwood Columbia Aging Project (WHICAP) and HRS is the “Health and Retirement Study”
