# Supplementary material for: Early life exposure to structural sexism and late‐life memory trajectories among black and white women and men in the United States
Source: Alzheimers Dement. 2024 Dec 18;21(2):e14410. doi: 10.1002/alz.14410 (PMC11848392; doi:10.1002/alz.14410)
Supplement: Supplementary file 5 — Supporting Information [file ALZ-21-e14410-s005.pdf]

1 SUPPLEMENTAL METHODS

1.1 Cohort Studies

The Washington Heights-Inwood Columbia Aging Project (WHICAP) is an ongoing study of community-dwelling Medicare recipients 65 years and older residing in Manhattan [1]. Recruitment occurred in three waves: 1992 (N=2126), 1999 (N=2180), and 2009 (N=2301). Participants completed a baseline assessment and were followed up at 18 to 24-month intervals for up to 25 years. During each session, participants were administered a neuropsychological battery and asked about their life-course social history, general health, functional ability and medical history. This study was approved by Institutional Review Boards at Columbia Presbyterian Medical Center, Columbia University Health Sciences, and the New York State Psychiatric Institute. Written informed consent was obtained.

The Health and Retirement Study (HRS) is an ongoing longitudinal nationally representative study of Americans aged 50 and older. The study was designed to examine the health, social, and economic factors associated with aging [2]. The initial cohort of participants (born between 1931-1941) started in 1992. A second cohort (individuals born before 1924) was added in 1993. In 1998, two additional cohorts (birth years between 1924-1930 and 1942-1947) were added to address age gaps and create a representative sample of individuals aged 50 and older. Since 1998, a new cohort of individuals aged 51-56 has been continually added to the HRS sample every 6 years. HRS oversamples Black and Hispanic/Latino/a/x/e (Latinx, hereafter) participants to improve reliability of estimates [3]. Participants are followed-up every two years to complete core interviews. These core interviews consist of content including (but not limited to): demographics, assets and income, physical conditions and treatment, health behaviors, cognitive function, among others [2]. Written informed consent was obtained from all participants in the

HRS study. Ethical approval for the HRS was obtained from the University of Michigan institutional review board.

1.2 Structural Sexism Measure

Our state-level structural sexism measure builds on a recently developed measure by Homan and colleagues [4]. This measure was designed to capture the degree of state-level sex/gender inequality in four domains: economic, political, cultural, and reproductive health. The current study is the first to use this measure to capture structural sexism from 1900 to 1960. The original measure was modified based on historical data availability and is described below. **Supplemental Table 1** provides information on data sources for each domain indicator. The economic domain is represented by ratios of men's to women's labor force participation, weekly earnings, and poverty rate (percent above the federal poverty line). The political domain is measured by the ratio of men to women occupying state legislature seats. The cultural domain aims to capture the extent to which ideologies that view women as subordinate to men influence the state-level environment [4]. Such ideologies are often espoused by religious conservative groups in the U.S., including Evangelical Protestants and Mormons [5]. Previous work has demonstrated the contextual influence of religious conservatism on state-level gender attitudes [6]. The percentage of a state population composed of religious conservatives was selected to measure this domain. Homan et al. [4] use the percentage of women in the state who live in a county without an abortion provider to measure the reproductive health domain. Given that data on abortion providers were limited or non-existent for most states between 1900-1960, the current study used state-level maternal mortality ratios for this domain.

Raw data for each indicator was extracted from publicly available data sources (see **Supplemental Table 1**). Ratios were created from aggregated data for each state and decennial

census year. Except for median weekly earnings and poverty rate, data were available for all indicators at each decennial year between 1900 and 1960. Data for median weekly earnings was available starting in 1940, and data for poverty rates was available beginning in 1950. Backcasting methods were used to impute values for these missing decennial years [7,8]. Estimated values for these indicators were consistent with available national data for the respective decennial years [9,10].

References

[1] Tang MX, Cross P, Andrews H, Jacobs DM, Small SA, Bell K, et al. Incidence of AD in African-Americans, Caribbean Hispanics, and caucasians in northern Manhattan. Neurology 2001;56:49–56.

[2] Sonnega A, Weir D. The Health and Retirement Study: A Public Data Resource for Research on Aging. Open Health Data 2014;2:e7. https://doi.org/10.5334/ohd.am.

[3] Fisher GG, Ryan LH. Overview of the Health and Retirement Study and Introduction to the Special Issue. Work Aging Retire 2018;4:1–9. https://doi.org/10.1093/workar/wax032.

[4] Homan P. Structural Sexism and Health in the United States: A New Perspective on Health Inequality and the Gender System. Am Sociol Rev 2019;84:486–516. https://doi.org/10.1177/0003122419848723.

[5] Chaves M, Eagle A. Religious Congregations in 21st Century America. National Congregations Study; 2015.

[6] Cotter D, Hermsen JM, Vanneman R. The End of the Gender Revolution? Gender Role Attitudes from 1977 to 2008. Am J Sociol 2011;117:259–89. https://doi.org/10.1086/658853.

[7] Jensen EB, Knapp A. Revisiting the Historical Estimates of Foreign-Born Immigration for the 2020 Demographic Analysis. 2016 Annu. Meet., PAA; 2016.

[8] Moahmed TA, El Gayar N, Atiya AF. Forward and Backward Forecasting Ensembles for the Estimation of Time Series Missing Data. In: El Gayar N, Schwenker F, Suen C, editors. Artif. Neural Netw. Pattern Recognit., Cham: Springer International Publishing; 2014, p. 93–104. https://doi.org/10.1007/978-3-319-11656-3_9.

[9] Derks S. The value of a dollar : prices and incomes in the United States, 1860-1999. Lakeville, CT: Grey House; 1999.

[10] Smolensky E, Plotnick R. Inequality and Poverty in the United States: 1900 to 1990. Madison, Wisconsin: Institute for Research on Poverty; 1993.
